# Nutritional Management in Liver Cirrhosis: A Combined Systematic Review and Observational Study

**DOI:** 10.3390/diseases13090278

**Published:** 2025-08-25

**Authors:** Valentina Amariței, Roxana-Elena Gheorghita, Olga Adriana Caliman Sturdza

**Affiliations:** 1 College of Medicine and Biological Sciences, Department of Biological and Morphofunctional Sciences, Stefan cel Mare University of Suceava, 720229 Suceava, Romania; valentina.amaritei@student.usv.ro; 2 College of Medicine and Biological Sciences, Department of Medical-Surgical and Complementary Sciences, Stefan cel Mare University of Suceava, 720229 Suceava, Romania; olga.caliman-sturdza@usm.ro; 3 Suceava Emergency Clinical County Hospital, 720224 Suceava, Romania

**Keywords:** diet, pathology, treatment, nutrition

## Abstract

Background: Liver cirrhosis is a complex and chronic pathology with the potential to impact a number of factors, including the patient’s health, nutritional status and diet. Proper nutritional intake plays an essential role alongside the necessary medical and recovery treatments. Methods: This study was conducted on a group that included patients of varying age demographics. They were required to undertake a 24 h food recall as well as two other questionnaires (CNAQ and CLDQ-NASH) that reported the level of appetite and nutrition and other aspects that focused on the patient’s general health and quality of life, respectively. Results: The results of the study indicated the presence of reduced appetite and a decrease in quality of life, as reported by questionnaire scores of less than 28 points for appetite and less than 4 points for quality of life. The 24 h dietary recalls revealed that the majority of patients exhibited a preference for meals comprising red and processed meats and traditional foods such as soups and animal foods and a low consumption of white meat, fish, legumes and fiber. Conclusions: The study’s findings reveal an imbalance in the patients’ nutritional intake and underscore the critical importance of nutritional support for patients with liver cirrhosis. However, further research is needed in this regard to determine the factors leading to nutritional deficiencies and the causes leading to refusal of nutritional intervention within the management of this disease.

## 1. Introduction

Liver cirrhosis causes a significant health burden all over the world since it has become the common health result of many long-lasting liver infections and illnesses and one of the leading causes of death. It has been estimated that the prevalence of chronic liver disease and cirrhosis is as high as 500–1100 per 100,000 people in Europe [1]. Causes of mortality owing to cirrhosis increased dramatically by 65 percent in the US between 1999 and 2016 [2]. Globally, cirrhosis ranks 11th in the leading causes of death (causing 2.4 percent of all deaths) and is a major cause of disease morbidity [3].

**Figure 1:**
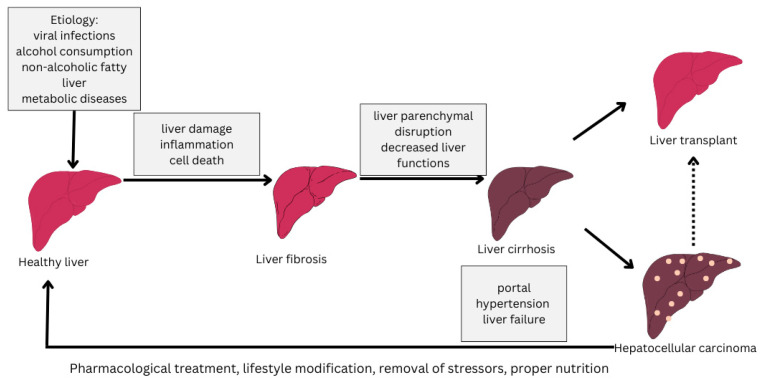
Stages of liver cirrhosis (adapted from [6]).

The progression of the disease varies from individual to individual but is influenced by abstinence from treatment and the influence of stressors such as alcohol consumption, inadequate nutrition or medication [4,5]. The stages of progression of cirrhosis of the liver from a healthy liver to cirrhosis of the liver are depicted in Figure 1.

Significantly, the landscape of cirrhosis etiology has changed over the past few years, whereby MASLD (NAFLD) has become one of the dominant causes [7]. As a result of this, most patients with cirrhosis are overweight or obese rather than underweight, but usually, the increase in weight is a marker of the underlying nutritionally-at-risk state, with skeletal muscle wasting and deficiencies in micronutrients (known as sarcopenic obesity) [8].

Malnutrition is one of the most important complications and prognosis factors in cirrhosis. The prevalence of its development is estimated at more than 50 percent of patients with advanced cirrhosis, although the reported prevalence ranges between 5% and 90% depending on the assessment method employed [9]. More importantly, malnutrition in cirrhosis is linked to significantly poorer clinical outcomes. Researchers have determined that malnutrition in cirrhotic patients increases the likelihood of hospitalizations and death by about 2 to 2.5 times in comparison with well-nourished patients [10]. Malnutrition is also a predisposing factor for severe complications like infections and hepatic encephalopathy, and it is frequently associated with sarcopenia and frailty, making the total number of problems even worse [11,12,13]. Consequently, malnutrition is no longer viewed as being at the periphery of hepatological disorders, but it is regarded in the latest medical literature as a direct complication of cirrhosis, thus requiring early recognition and management [9]. This is to say that nutritional status should be equally considered alongside the management of portal hypertension and other traditional complications if we want to better the prognosis for patients.

The malnutrition of liver cirrhosis has a multifactorial pathophysiological basis. Liver is the key organ for nutrient processing (storage of glucose, synthesis of protein, absorption of fats through bile secretion, etc.); therefore, chronic liver failure disarranges the processes on a number of levels [8,14]. Poor dietary intake is a key factor; many cirrhotic patients exhibit poor food intake secondary to early satiety with ascites (fluid pressure on the stomach), slow emptying of foodstuff (gastric retention), distortion of flavors and smells (in part a result of zinc and vitamin A deficiencies) and nausea, or they face dietary restrictions (e.g., low sodium diets to reverse ascites) [15,16,17,18]. There is also frequent malabsorption of nutrients (particularly fat and fat-soluble vitamins) because of poor bile acid secretion, overgrowth of the small intestine with bacteria, or pancreatic failure in alcohol-related diseases [8,19,20]. Moreover, cirrhosis also results in a hypermetabolic, catabolic state: there is a decline in hepatic glycogen stores and insulin resistance that results in an increase in starvation metabolism even during only brief fasting and a breakdown of protein sources (muscle) to provide gluconeogenic substrates [21,22,23,24,25,26]. Hormonal disturbances (such as the occurrence of elevated circulating levels of ghrelin, which, ironically, does not induce appetite) subsequently worsen the effects of muscle wasting and metabolic imbalances [27]. This process is also considered multifactorial and has been linked to an imbalance in the gut microbiome [8].

Since malnutrition is quite common in cirrhotic patients and has significant clinical consequences, providing early nutritional interventions would be beneficial. The argument for earlier intervention is that waiting until a patient is already suffering from severe protein–calorie malnutrition and loss of muscle mass is likely to worsen clinical outcomes, as the intervention targets an entrenched clinical risk factor in this patient group. New evidence indicates that proactive nutrition therapy (provided by a multidisciplinary team) is capable of enhancing both the survival rate and quality of life in patients with cirrhosis [28,29]. As a result, the current clinical guidelines reflect the importance of nutritional care in managing cirrhosis. As an example, both the European Association for the Study of the Liver (EASL) in 2019 and the American Association for the Study of Liver Diseases (AASLD) in 2021 issued guidelines calling for every cirrhotic patient to be screened regularly to identify malnutrition and initiate nutritional support based on the risk or deficiencies identified [8,9]. In short, current approaches to treating cirrhosis consider optimal nutrition a crucial component rather than auxiliary care.

Nutritional approaches to cirrhosis aim to address both macro- and microelements, meal timing, and macronutrient and micronutrient requirements to remedy malnutrition. The contemporary clinical recommendations are as follows:

- Sufficient Energy and Protein: A high-calorie, high-protein diet should be used to prevent or reverse protein–energy malnutrition. Standard goals are 30 to 35 kcal/kg/day of energy and 1.2 to 1.5 g/kg/day of protein (or higher in nutritionally at-risk patients) across multiple meals per day [30]. Protein restriction has also now been discouraged in any patient with hepatic encephalopathy; patients with cirrhosis (including those with encephalopathy) should be given at least 1.2–1.5 g/kg of protein per day as tolerated to maintain nitrogen balance [9]. Protein is vital to the maintenance of muscle mass, and issues related to the exacerbation of encephalopathy due to high protein intake are addressed with drugs (i.e., lactulose), rather than decreasing protein intake, according to current recommendations [31,32].
- Meal Timing and Meal Frequency: Meals should be small and frequent, and prolonged periods of fasting are not recommended [33,34,35,36]. Patients with cirrhosis are advised to receive 3–5 meals with 2–3 snacks in a day, with 1 of them in the late evening consisting of carbohydrates [37]. Moreover, 50 g of complex carbohydrates as a bedtime snack is advised to provide a source of energy while the patient is asleep, as they may not eat food overnight. This approach was demonstrated to ameliorate sarcopenia in cirrhosis, enhance lean body mass, and improve nitrogen status [24]. Generally, frequent feeding (punctuated only by a late-night snack) allows one to minimize fasting-induced muscle catabolism, which makes it an easy yet effective dietary change [9].
- Sodium Control: A mild sodium restriction diet is normally suggested in patients with ascites since it can help control fluid retention. Guidelines frequently recommend the use of a no-added salt diet (approximately 56 g of salt daily [38]). Nevertheless, sodium restriction must be considered together with palatability and overall intake, as overly strict limits can render food unattractive, decreasing food intake and further exacerbating malnutrition [39]. Clinicians are advised to personalize sodium intake limits to reduce ascites without unnecessarily reducing patients’ nutritional intake.
- Oral Nutritional Supplementation: If patients are unable to receive the required proportion of calories or protein via an oral diet, oral nutritional supplements (ONSs) must be introduced. Add-ons (like high-protein, energy-rich oral supplements) have been demonstrated to considerably restore body composition (increase lean mass and BMI) and the serum proteins of nutritionally at-risk cirrhotic individuals [40,41]. A meta-analysis showed that oral supplementation can be used to enhance the clinical outcomes of patients with cirrhosis [42]. Of particular interest is taking supplements in the late evening (nighttime ONSs), which has been found to result in a more beneficial protein status than daytime supplementation, aligning with the prioritization of late evening feeding [43,44]. On the whole, ONSs play a beneficial and supportive role in meeting nutritional outcomes, and they are usually fortified with vitamins and minerals to remedy micronutrient deficits.
- Micronutrient Repletion: Vitamin and mineral deficiencies are commonly found in cirrhotic patients, with levels of vitamin D, zinc, magnesium, and fat-soluble vitamins (A, E, K) often being low as a result of malabsorption and/or the effects of chronic disease [45,46,47,48,49]. Micronutrient deficiencies must be identified and treated. Practice guidelines recommend supplementing any confirmed deficiencies, or those with strong evidence, with the related vitamins or minerals [9]. As an example, vitamin D levels should be assessed, as a majority of the population is deficient, and vitamin D supplementation should be provided in the case of a vitamin deficiency (<20 ng/mL) until sufficient levels are achieved [50]. Another significant micronutrient is zinc. Supplementation is frequently advised for patients with a zinc deficiency or breakthrough hepatic encephalopathy; it is possible that increasing zinc levels can boost both ammonia metabolism and taste performance (and ideally appetite) [50]. In general, although routine mega-dose supplementation is not recommended without evidence of deficiency, supplementation to restore nutritional deficiencies is an important part of comprehensive care [29].
- Branched-Chain Amino Acids (BCAAs): BCAAs (leucine, isoleucine, and valine) are essential amino acids that are usually deficient in people with cirrhosis because of the disturbed metabolism of amino acids and their utilization as alternative sources of energy [51,52]. BCAA supplementation has been shown to have a number of potential benefits in advanced liver disease: BCAA supplementation has the potential to prevent muscle protein breakdown, improve nitrogen balance, enhance muscle mass, limit the development of complications, and improve quality of life [53,54,55]. Clinical studies have also reported that the neuropsychiatric performance of patients with hepatic encephalopathy is improved with BCAA supplementation, probably due to the substitution of an alternative nitrogen source and the detoxification of ammonia [56]. BCAA-enriched nutritional supplements are specifically recommended for patients with decompensated cirrhosis or those who are unable to consume enough protein due to encephalopathy or other problems [57]. This may result in the use of nighttime BCAA drinks or increasing the portion of vegetable protein (which is naturally higher in BCAAs and less likely to produce ammonia) in patients with refractory hepatic encephalopathy [58]. In this way, BCAA supplementation is a scientifically justified intervention to sustain protein intake and muscle anabolism in nutritionally compromised cirrhotic patients.
- Enteral Nutrition Support: Inadequate oral feeding, even after dietary advice and use of ONSs, may necessitate the use of early enteral tube feeding to achieve nutritional goals. Gastrointestinal nutrition (e.g., through a nasogastric or nasoenteric feeding tube) is mostly safe and well-tolerated in patients with cirrhosis; in fact, the absence of non-bleeding esophageal varices, even when a nasogastric feeding tube is to be inserted, is not indicative of contraindication [59]. Elective endoscopic gastrostomy tubes are, however, riskier in advanced disease and should normally be averted [60]. Early enteral nutrition in nutritionally at-risk cirrhotic patients is presumed to stabilize energy and protein intake, prevent further muscle loss, and enhance clinical outcomes. This feeding method is used instead of parenteral nutrition when the gastrointestinal tract is in working order, as it reduces the risk of infection and delivers nutrients in a more physiologic manner. Parenteral nutrition is used only in exceedingly unique cases when enteral nutrition cannot be carried out (such as in intestinal failure or protracted ileus) or when oral/enteral nutrition cannot be withheld longer than 72 h [59,61]. In these cases, close follow-up is advised since infections (e.g., central line IV infections) and metabolic problems can occur in cirrhotic patients. Thus, early use of enteral nutrition in individuals with the inability to receive oral nutrition is an essential measure to avoid starvation and treat malnutrition in this at-risk group [62].

In conclusion, proper nutrition is a major branch in the treatment of patients with liver cirrhosis, as most are nutritionally at-risk, and malnutrition has a pernicious effect on patients’ clinical outcomes. An integrative, clinical evidence- and guideline-based strategy comprising optimal nutrition (calories/protein), meal timing, micronutrient correction, and nutritional support (oral or enteral) would reduce the pathophysiological effects of malnutrition in patients with cirrhosis [28,29]. In patients with malnutrition, early and aggressive treatment allows clinicians to improve the strength of patients, decrease complications, and potentially increase survival, which is why nutritional therapy is considered a central tenet in the comprehensive treatment of cirrhotic patients.

## 2. Materials and Methods

This study aimed to analyze the role of nutrition in the management of patients with cirrhosis of the liver in the “Infectious Diseases” ward of the “St. John the New County Emergency Clinical Hospital” in Suceava. This study was observational and included a group of 50 patients, the majority of whom were female (39 F and 11 M), aged between 29 and 71 years. They were selected for participation in this study based on the presence of a liver cirrhosis diagnosis and being hospitalized in the respective hospital ward.

Prior to the start of the study, patients signed an informed consent form containing general information on the pathology under study, the aims and objectives of the research, the methodology used, the implications of their participation, and details on data confidentiality. This study was approved by the Ethic Committee of “St. Ioan Cel Nou,” Suceava, Romania, with approval code 81, on 18 December 2024.

The first questionnaire, entitled the Council of Nutrition appetite questionnaire, consisted of a set of questions assessing multiple aspects of appetite, such as changes in hunger, early satiety, perception of pleasure and taste associated with eating, the number of meals consumed per day, the presence of nausea, and patients’ general mood. This tool was used to assess the quality of appetite among the patients who participated in the study and plays a primary role in nutritional risk assessment. Quantification of appetite is performed using different items, with each letter representing a number (a-1, b-2, c-3, d-4, e-5). A number lower than or equal to 28 points represents a significant risk of poor appetite, i.e., unintentional weight loss of at least 5 percent within six months. The questionnaire was adapted into Romanian according to the methodology applied in the article “Appetite assessment: simple appetite questionnaire predicts weight loss in community-dwelling adults and nursing home residents” [63].

The second questionnaire used was the 24 h dietary recall, which was applied to assess the nutritional intake of patients with cirrhosis of the liver. This method involves the detailed recording of all food and beverages consumed by the patient in the last 24 h, providing a clear and easy way to assess the frequency of meals and snacks as well as the diversity of food groups in patients’ diets. Also, this prospective questionnaire aimed to identify possible nutritional imbalances, as most patients with cirrhosis are nutritionally at-risk, check dietary compliance according to nutritional guidelines and recommendations, and evaluate food preferences and cooking methods. In order for patients to accurately report the portions and grams, they were provided with pictures and tabulated representations of how much a portion represents, according to the food group, in accordance with the “Healthy Eating Guide” [64] developed by the “Romanian Nutrition Society”. Patients were also provided a model to help them use the tool and educate them on the importance of healthy eating. This questionnaire was translated and adapted into Romanian according to “2.14 NACS USER’S GUIDE MODULE 2-Nutrition Assessment and Classification, How to do 24-Hour Recall” from the “Food and Nutrition Technical assistance III Project (FANTA)”.

The last questionnaire, the CLDQ-NASH (Chronic Liver Disease Questionnaire for Nonalcoholic Steatohepatitis), was applied to measure the impact of liver cirrhosis on quality of life and is sectioned into different categories: abdominal level symptoms (discomfort, feeling of fullness), sleep quality (insomnia, fatigue), energy level, muscle strength, appetite, specific symptoms (fever, shortness of breath, weight loss, pruritus), ability to perform daily activities of daily living (climbing stairs, walking), psychological level symptoms (anxiety, depression), and personal concerns related to the pathology (concerns about the impact of the disease on family members, disease progression). The patients answered each question, with a total of 36 items, using a Likert scale for the interpretation of the answers (from 1 “Always” to 7 “Never”), to indicate the impact of the pathology and associated symptoms on the health and diet of the patient with cirrhosis of the liver. This questionnaire was adapted and translated into Romanian according to the article “Validation of Chronic Liver Disease Questionnaire for Nonalcoholic Steatohepatitis in Patients with Biopsy-Proven Nonalcoholic Steatohepatitis” [65]; however, it was applied in the aforementioned study to analyze the impact of the disease in people with nonalcoholic steatohepatitis, a condition with similar symptoms and with an increased risk of progression to cirrhosis of the liver.

At the end of the questionnaires, the patients were given a set of nutritional recommendations containing information on adequate calorie and protein intake, indicated and contraindicated foods, and gastro-techniques. The nutritional recommendations aimed to educate and raise patients’ awareness of the importance of nutrition in the management of this complex pathology. The information was processed and adapted according to the “ESPEN practical guideline: Clinical nutrition in liver disease” [32] and “Nutrition for healthy and sick people (recommendations for the most diverse cases of disease)” [66].

The questionnaires were not forward- or back-translated. Data was collected utilizing Microsoft Excel 2010 software, while statistical analysis was conducted using Origin Pro version 2024 (OriginLab Corporation, Northamptom, MA, USA). The r correlation coefficient was evaluated using R studio (version 4.5.0), with the statistical interpretation being facilitated using ANOVA, Pearson’s coefficient, and Spearman’s correlation coefficient.

## 3. Results

A total of 50 patients participated in this study, aged between 29 and 71 years (Table 1), of whom 35 (70%) were female and the remaining 15 (30%) were male. All patients presented the presence of liver cirrhosis as the main marker, with most presenting an infectious etiologic factor (liver virus type B or C), since this group was recruited from the infectious ward of an emergency hospital in Suceava.

According to their weights and BMI calculation, most patients were nutritionally at-risk (2 normal weight, 22 overweight, 22 with grade 1 obesity, and 4 with grade 2 obesity), confirming that they had difficulty in maintaining a proper diet; however, this may also have been caused by the presence of ascites among the patients (Table 2).

**Figure 2:**
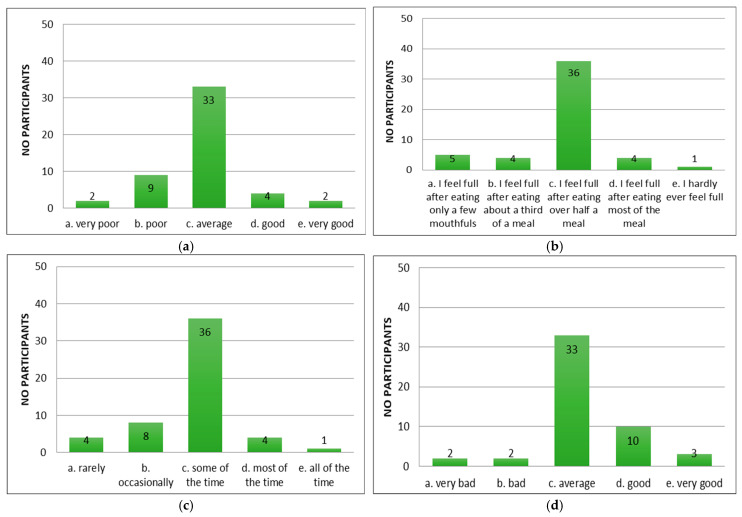

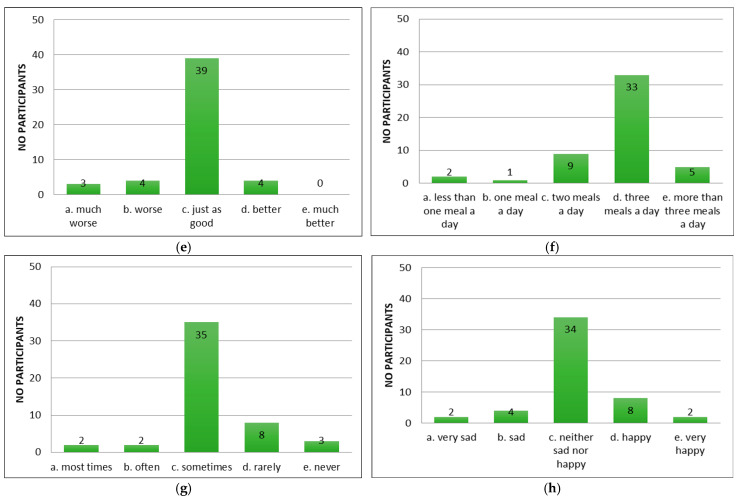
Evaluation of appetite in patients using CNAQ items. (**a**) Types of patient appetite. (**b**) Patient-reported post-meal feelings. (**c**) Self-reported hunger frequency. (**d**) Patients’ taste perception of food. (**e**) Changes in taste perception of food over time. (**f**) Usual number of meals per day. (**g**) Frequency of nausea or sickness during eating. (**h**) General mood as reported by respondents.

Following the application of the appetite questionnaire “CNAQ”, the majority of the patients with cirrhosis of the liver confirmed that their appetite was average (Figure 2a). Patients reported feeling full after eating more than half a meal (Figure 2b) and feeling hungry some of the time (Figure 2c), but that food tasted average (Figure 2d). Compared to the period when they did not have cirrhosis of the liver, they considered food to be just as good (Figure 2e). Most of them consumed three meals per day (Figure 2f). Patients reported occasionally feeling nauseous while eating (Figure 2g) and a mixed psycho-emotional state (Figure 2h).

**Figure 3:**
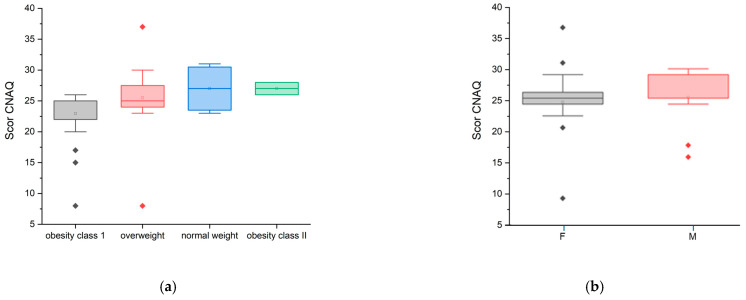
CNAQ scores by weight status categories (**a**) and sex (**b**); 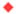
—statistical outliner for female group, 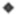
—statistical outliner for male group.

In the CNAQ’s score calculation (95% confidence interval [CI]: 2.00 ± 0.22), the majority of the group represented an average score below 28 points, which confirms that they present a low appetite and possible malnutrition, i.e., involuntary weight loss in the last five months. The lowest score was 8 points, and the highest score was 37 points. Although the mean CNAQ scores across BMI categories (normoponderal, overweight, obesity grade I and II) varied slightly between groups, the differences were not statistically significant (ANOVA, *p* = 0.172) (Figure 3a). Furthermore, no statistically significant difference was observed between female and male participants (ANOVA, *p* = 0.580) (Figure 3b).

Statistically, the Pearson correlation was significant (coefficient (r) = 0.3017, *p*-value = 0.0332, *p* < 0.05), establishing a weak to moderate positive correlation between the “CNAQ” and the “Weight” variable. Also, the Spearman correlation was slightly stronger compared to the Pearson correlation, which is evidenced by the fact that there is a positive relationship between the “CNAQ” score and the “Weight” variable. It can be concluded that the “CNAQ” score increases slightly as the Weight score increases, indicating that people with a higher Weight score tend to have a better appetite (Figure 3a). Also, the “CNAQ” score is higher in male patients compared to female patients (Figure 3b).

According to the documented times of the primary meals, the designated breakfast interval was from 7 a.m. to 12 noon, the lunch interval was from 12 noon to 6 p.m., and the dinner interval was from 5 p.m. to 9 p.m. A majority of the study participants consumed all three main meals (40 participants), a smaller number consumed two meals (n = 9), and one participant consumed only one meal.

**Figure 4:**
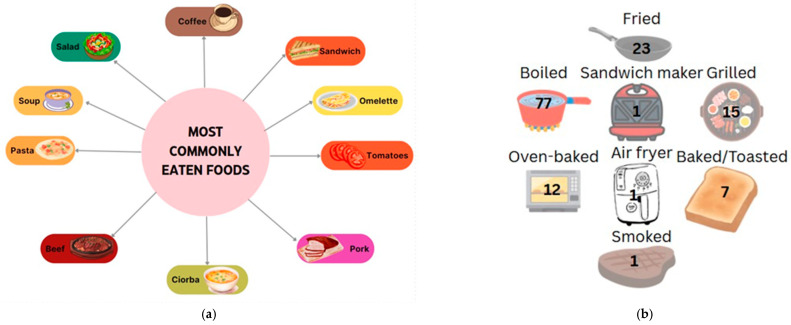
Most preferred foods (**a**) and their main cooking methods among study participants ((**b**)—number mentioned represent the number of persons that chose the option).

Data collected from patients who responded to the 24 h recall showed a preference for eating vegetables, fruit, dairy products, cereal products (especially bread and porridge), and traditional foods such as soups. For main meals, the most frequently reported foods in participants’ diets were steak, sandwiches, omelets, yogurt, and soups. Dairy, eggs, and bread were among the most common food choices. In terms of fruits and vegetables, tomatoes and pears were the most frequently mentioned, often eaten in salads or sliced, indicating a preference for raw rather than cooked options. Other fruits and vegetables, such as eggplant and bananas, were rarely consumed, although they are important nutritional options in the context of a hepatoprotective diet. In terms of cooked vegetables, mashed potatoes and legumes, such as pods, were preferred. In terms of the cereal products, pasta, bread, and porridge in various combinations were the most preferred. There was also a reluctance to consume fish or less traditional foods such as avocado and muesli. When comparing protein sources, it was found that patients preferred to consume pork and beef (steak, ribs, chops) rather than white meat, chicken, or fish, indicating a preference for red meat and those high in saturated fat. Another observation is the frequent consumption of coffee in the studied group; however, coffee is a hepatotoxic food and should be avoided, along with foods such as pickles, cold cuts, and fried foods, which were also reported by the participants (Figure 4a).

In terms of gastronomic techniques, cooked dishes are preferred, with this method being mentioned by patients 77 times. Roasting is another preferred method (23 mentions), but it is less recommended for patients with cirrhosis of the liver due to high fat intake and the possible induction of abdominal discomfort, pain, or a burning sensation in the esophagus. Other methods were reported by the group, such as grilling and baking (13 and 12 cases, respectively), which are healthy alternatives to frying; however, the ingredients used, such as spices or sauces, are of particular importance. The least mentioned methods were smoking or the use of various tools such as a sandwich maker or air fryer, which were only reported by a small number of patients (Figure 4b).

The snacks most frequently consumed by the group, according to the food recall questionnaires collected, were fruits (such as bananas and apples) and yogurt, which are simple, easily digestible options that are rich in carbohydrates and, in the case of yogurt, also high in protein. Other foods that were seldom reported but present a complex nutritional profile are combinations such as sheep’s cheese with vegetables or dishes combined with tomatoes or bread. Additionally, the group occasionally consumed foods with high caloric density and low nutritional value, such as hot chocolate, pretzels, and biscuits, which may lead to an unbalanced energy intake and symptom intensification. Another observation is that this sample (n = 11) preferred eating raw foods as snacks, although they also consumed baked and cooked foods. The consumption of raw foods, especially fruits, is beneficial for micronutrient and fiber intake, but can pose a risk, especially if consumed in excess, for patients with cirrhosis of the liver or those experiencing hepatic encephalopathy or abdominal discomfort.

Although this group reported eating snacks, most did so infrequently (n = 36), and only three of them ate a snack in the evening, which is an important recommendation according to nutritional guidelines.

According to the 24 h recall, the average energy intake was 1310 ± 439, 53 kcal. The lowest energy intake was 459 kcal (female, 29 years old), and the highest was 2224 kcal (male, 70 years old). Regarding protein intake, the average value was 60 ± 1.41 g, with the lowest value being 12.40 g (male, 55 years old) and the highest being 133.20 g (male, 61 years old). The mean fat intake was 56.16 ± 28.40 g, with a minimum of 12 g and a maximum of 126.40 g. The average value of carbohydrate intake was 118.14 ± 17.82 g (with 18.40 being the lowest value and 274.40 being the highest). According to nutritional guidelines, for a normal-weight person (approximately 70 kg) with cirrhosis, the number of calories should be between 30 and 35 kcal/kg body weight (~2100–2450 kcal), and protein intake should be 1.2–1.8 g/kg body weight (~84–126 g) [67].

All calculations and quantities were adapted using the Nutrition Value app [67] and Carbs & Cals Carb & Calorie Counter 6th Edition [68]. Some quantities were estimated using the book mentioned [68] if patients did not mention the weight of their food; when the weight was specified, the quantities were calculated based on the reported measurements.

The most representative example of an appropriate dietary pattern, according to nutritional guidelines, was a 56-year-old patient. She indicated in her dietary report the consumption of three main meals and three snacks, including one late in the evening, aligning with the guidelines. Breakfast is an omelet and two slices of bread; the first snack is two slices of cheese with tomatoes, cucumber, and peppers; lunch is chicken soup; the second snack is an apple; dinner is turkey with boiled potatoes; and the last snack, eaten at 20:30, is yogurt with fruit. This diet included foods with animal protein and white meat and was rich in vegetables, fruits, fiber, dairy, and cereal products. Although some of the foods described by the patient may lead to complications if consumed in excess (such as tomatoes and apples), this diet is appropriate for a person with cirrhosis of the liver. Also, the preparation methods used, such as gentle cooking, support the patient’s digestion and are an important recommendation from a gastro-technical point of view. A nutritionally non-conforming example is from a 62-year-old participant. Her diet consists of three main meals with no snacks. For breakfast, she eats yogurt with a banana; for lunch, mushroom and garlic stir-fry and roast pork; and for dinner, potatoes and a fried egg. In terms of meals, she does not eat 4–6 meals, nor an evening snack, as recommended by the guidelines. Breakfast meets the guidelines, but the lunch, containing saturated fats, pork, garlic, and sauteed mushrooms, all prepared by frying, is not recommended. The dinner also consists of fried food, three eggs, and two medium-sized potatoes, resulting in a high caloric and lipid intake, potentially leading to digestive and metabolic difficulties for patients with cirrhosis of the liver. This dietary pattern contradicts the guidelines, which recommend limiting saturated fats, fried foods, and potentially hepatotoxic preparations.

**Figure 5:**
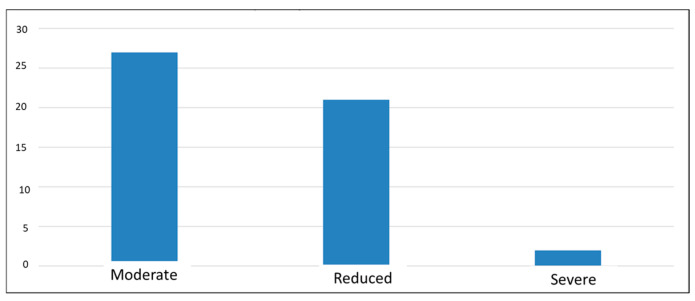
Classification of symptom severity according to CLDQ-NASH score.

The results of the last questionnaire administered, the “CLDQ-NASH”, indicate a decrease in quality of life, with average scores of 3–4 (out of a maximum of 7) for most participants, suggesting that they experienced changes in most of the areas analyzed. The lowest score was 1, and the highest was 6.19, which may emphasize that in this complex pathology, each individual experiences symptoms differently, i.e., a diverse perspective regarding the influence of the disease on their health (Figure 5).

**Figure 6:**
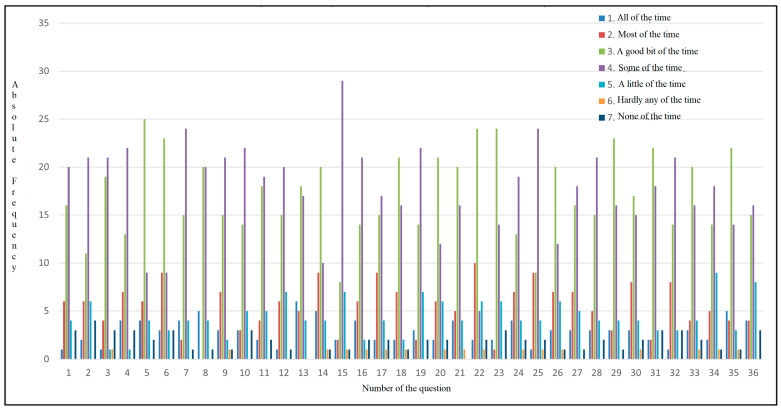
Absolute frequency distribution for CLDQ-NASH responses.

As shown in Figure 6, each answer is associated with a color (1 dark blue, 2 red, 3 green, 4 purple, 5 light blue, 6 orange, and 7 navy blue), and the absolute frequency represents the number of participants who most frequently selected each answer. The majority of the participants reported experiencing bloating, fatigue, body pain, and sleepiness during the day, with all these symptoms occurring some of the time. Out of 50 participants, 25 of them had abdominal pain a good part of the time. The patients also had shortness of breath, which was a problem for carrying out daily activities. As for eating, 24 participants reported that they sometimes cannot eat as much as they want, and 20 participants reported experiencing muscle weakness. The majority of them reported having difficulty carrying heavy objects, feeling anxious, having low energy levels, sometimes feeling unhappy, and feeling sleepy during the day. Their diet is limited, with 20 of them indicating that a good part of the time, they do not consume what they want or cannot because of the symptoms they experience, which may indicate a possible cause of nutritional imbalance and also a loss of appetite. In total, 29 participants answered that sometimes, they feel irritable, have difficulty sleeping at night, and experience abdominal discomfort.

This pathology affects not only the patients, but, from their perspective, also their family, as confirmed by the responses indicating that the disease sometimes worries them and can influence their social environment. Many of them indicate that they have difficulty falling asleep and experience frequent muscle cramps. Concerns about worsening symptoms are evident, with 24 of them reporting they feel concerned a significant part of the time. Additionally, a majority of the sample (n = 24) reported experiencing dry mouth. Changes are also observed on the psycho-emotional side, with patients sometimes feeling depressed and worried about their health worsening; 20 participants indicate that they have difficulty concentrating a good part of the time. Other symptoms confirmed to be intensified were pruritus, decreased energy and performance of daily activities, stress, joint pain, and lethargy during the day (Figure 6).

**Figure 7:**
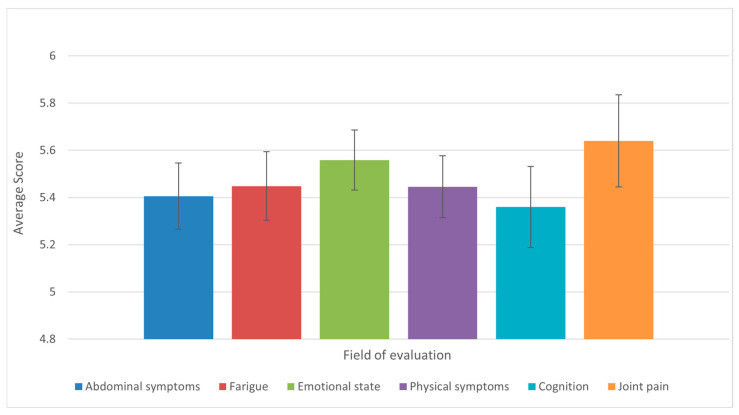
Average scores across the six CLDQ-NASH domains among patients with liver cirrhosis (n = 50). Bars represent means, and error bars indicate standard error of the mean (SE). Domains with lower average scores reflect greater symptom burden and impaired quality of life.

Analysis of the results of the 36-question questionnaire found that the domains of joint pain and mobility, emotional states, physical symptoms (appetite, fever, shortness of breath, weight loss, pruritus), and fatigue were the most affected, with the same mean score observed. Conversely, the domains of abdominal symptoms and cognition were found to be moderately affected (Figure 7).

## 4. Discussion

The present study aimed to highlight the importance of nutritional management in patients with cirrhosis of the liver, which was demonstrated by the responses received from patients, following the completion of the 24 h dietary recall and the appetite and quality of life questionnaires (“CNAQ” and “CLDQ-NASH”). To emphasize the results of the study and confirm their relevance, the most important nutritional guidelines applied for this pathology were reviewed, comparing them to the diets described in the recall and important scientific articles reporting the application of the tools used in this study. In this regard, according to the “ESPEN” and “EASL” guidelines on the nutrition of patients with liver cirrhosis, patients should follow a diet rich in quality protein, mainly from lean animal sources (such as chicken, white fish, and eggs) and plant sources (such as legumes and dairy products). The guidelines also emphasize the importance of including a late evening snack to prevent nocturnal protein catabolism. From a gastro-technical point of view, boiling food is in line with the recommendations of the ESPEN and EASL guidelines, as it favors high digestibility and reduces added fat content, and is suitable for patients with liver cirrhosis. The “AASLD” guidelines recommend limiting the intake of saturated fat and red meat (such as pork and beef) and increasing the intake of fiber, omega-3 fatty acids, and antioxidants through the consumption of fatty fish, nuts, and green vegetables. However, the data collected from the 24 h recall showed that patients in the study preferred eating pork, while white meat (chicken and fish) was reported much less frequently. Also, foods high in fiber and unsaturated fatty acids (e.g., trout, eggplant, almonds, and bananas) were only reported occasionally or not at all. This suggests a possible unbalanced diet, which may increase the risk of protein–energy malnutrition, exacerbate symptoms such as hepatic encephalopathy and ascites, and contribute to disease progression. Another observation is that most of the participants did not consume a late evening snack, with only three doing so. Also, the patients used boiling as the predominant cooking method, which attests to compliance with one of the primary recommendations in the nutritional guidelines for these patients.

Wang and Shen [69] used a more simplistic version of the “CNAQ”, referred to as the SNAQ, in a sample of 70 elderly patients with cirrhosis of the liver, recruited from a hospital in China. Similar to the “CNAQ”, the purpose of the questionnaire is to identify the appetite level and nutritional risk of this category of patients; the highest score is 20, and a score below 14 points is considered an associated risk, making it much easier to interpret. Thus, 17 patients had a score above 14 points, and 53 had a score below 14 points. The increased number of patients with a score below 14 points emphasizes the presence of poor appetite and a high risk of weight loss and malnutrition. The symptoms reported in most cases were a lack of appetite, decreased satiety, and a decrease in the pleasure of eating. Those who scored low on the questionnaire were also found to have a lower BMI compared to normal values (a BMI less than 18.5 kg/m^2^). Moreover, an SNAQ score below 11.5 represented a key point for the identification of significant weight loss, more than 5% in the last 6 months [69]. The study conducted by Rudzinska et al. [70] used the “CNAQ” as an appetite interpretation questionnaire in a group of elderly patients with liver pathologies who were over 65 years of age; however, it was not accurately confirmed whether they had liver cirrhosis. Application of this questionnaire in the described group revealed appetite disturbances and their influence on the patients’ diet and the evolution of their symptoms. The mean score following the application of the questionnaire among the group was 27 points (a score of less than 28 points represents low appetite), and 62.9% of the patients had appetite disturbance. This study also states that liver diseases can affect taste perception and thus lead to changes in appetite, with the main causes being the production of factors that contribute to the intensification of bitter or unpleasant tastes in the mouth and damage to the taste pathways in the brain [70]. In a British study conducted in 2024, the researchers implemented the appetite questionnaire and 24 h recall in a group of 20 patients presenting with liver cirrhosis, with or without the presence of hepatic encephalopathy. The 24 h recall showed that patients with cirrhosis and hepatic encephalopathy consumed twice as much as those without hepatic encephalopathy (216 g versus 119 g, *p* = 0.023), but both groups did not reach the daily nutrient and protein requirements according to the nutritional standards of 25–35 kcal/kg and 1.5–2 g/kg, respectively [71].

In the KIRRHOS study carried out in 2021, according to the application of 24-hour boosters to patients diagnosed with liver cirrhosis, it was confirmed that energy intake decreases in parallel with the “EALS” and “ESPEN” guidelines, and protein intake was insufficient. It was observed that the group opted for a diet high in fat and sugar, with insufficient dietary fiber intake. In terms of food groups, the patients consumed small amounts of whole grains, legumes, fruits, poultry, and fish, preferring the consumption of red meat and foods high in sugar. The average number of meals consumed by them was between three and four meals per day, and a small number of them consumed a late evening snack (17%). Also, in the group of patients with refractory ascites, their energy and protein intakes were high compared to the overall sample but were within the recommended limits of the nutritional guidelines analyzed [72]. In the study conducted in 2021 by P. Sharma et al., the authors analyzed the nutritional status of patients with liver cirrhosis using anthropometric, clinical, and laboratory data and 24 h recall and food frequency questionnaires, according to the stage of liver cirrhosis determined via the Child–Pugh score. Thus, the entire group of participants had protein and calorie intakes below the guideline-recommended levels. Most of them had three meals a day, but some reported having two meals, three meals and one snack, or three meals and two snacks a day; late evening snacking was not common among patients with liver cirrhosis. The majority of subjects were lacto-vegetarians (61%), and the remaining were non-vegetarians (39%), indicating adherence to the consumption of plant foods and avoidance or moderate consumption of animal foods. Participants’ appetite was low, accompanied by symptoms such as reduced taste perception (likely due to opting for low-sodium diets), feeling of fullness, abdominal distension, and vomiting [73]. In 2023, Pham and Nguyen conducted a study in which they used 24 h dietary recall as a tool to assess the dietary intake of patients with liver cirrhosis. The measurements showed that energy and protein intake were below the guideline-recommended levels, about 22.4 kcal/kg per day of energy and 1.1 g/kg/day of protein. They compared the collected data to the “ESPEN” guidelines. The majority of participants did not meet the daily requirements for energy (78.4%), protein (45.1%), and dietary fiber. Micronutrient deficiencies were also identified (vitamin A, B complex, calcium, magnesium, iron, and zinc), demonstrating a nutritional imbalance, which is frequently encountered in this category of patients; thus, nutritional intervention is of particular importance [74]. In the study conducted in 2024 by Ferreira et al., the authors used a 24 h booster for liver cirrhosis patients on the liver transplant waiting list, with the intervention taking place over twelve weeks. The results of the study showed that energy and protein intake were below the nutritional recommendations, with approximately 25.5% meeting the minimum energy requirement of 30 kcal/kg/day and 36.2% meeting the protein requirement of 1.2 g protein/kg/day. Consumption of an evening snack was relatively low (44%), and main meals were high in sugars and saturated fatty acids, with a low intake of fiber, legumes, fruits, vegetables, and white meat. Also, these patients preferred foods high in fat and sugar, processed foods, and red meat, which, according to nutritional guidelines, should be avoided or consumed moderately [75]. In another study conducted by Nguyen with collaborators, they evaluated 40 patients with cirrhosis of the liver, also using the 24 h dietary recall to determine the daily protein and energy intake. The mean energy intake was approximately 24.6 kcal/kg body weight/day, and the mean protein intake was approximately 1 g/kg body weight/day, both below the recommended levels of 30–35 kcal/kg and 1.2–1.5 g protein/kg/day, respectively, according to international nutritional guidelines. Also, 32.5% of patients were eating at least four meals a day; 22.5% included a late evening snack in their diet, which is an important recommendation according to current guidelines for those with cirrhosis of the liver; and 40% avoided eating animal protein, which may contribute to lower protein intake. Consumption of alcohol, animal protein, and fats or oils was low, with 67.5%, 40%, and 35% of patients not consuming them, respectively. Plant proteins were mainly consumed (95%), which may highlight either a possible reluctance to animal proteins due to hepatic encephalopathy or a lack of appropriate nutritional counseling [76].

A 2018 study analyzed the quality of life of more than 149 patients diagnosed with cirrhosis of the liver, using the “CLDQ” as a validated instrument. These patients were divided into three categories according to the severity of the disease determined via the “Child–Pugh” classification (A, B, and C). The results of the questionnaire were interpreted, and the mean total score was statistically significant, decreasing with the severity of cirrhosis, with a lower score also representing a decrease in quality of life. The most affected areas, according to the data from the questionnaire, were as follows: fatigue, systemic symptoms, emotional state, and abdominal symptoms, emphasizing a major deterioration in the physical and mental status of these patients [77]. In 2019, researchers applied the “CLDQ” to 72 patients diagnosed with liver cirrhosis, aiming to assess nutritional knowledge and monitor symptoms occurring in this pathology. After the nutritional intervention, at 12 weeks, an increase in the CLDQ scores was reported, from 4.22 to 7.1, with a *p* < 0.0001, emphasizing the importance of nutrition in these categories of patients. The symptoms analyzed, such as fatigue, decreased appetite, and feeling of fullness, showed improvements that were beneficial changes for the patients. The improvement of symptoms after nutritional intervention emphasizes that an appropriate diet following guideline recommendations, along with continuous monitoring of the patient’s health status and appropriate medication, can help patients with liver cirrhosis improve their quality of life and prevent symptom progression [78]. In a 2020 study, researchers administered the CLDQ-NASH to patients with non-alcoholic steatohepatitis and cirrhosis of the liver. The mean score reported among participants was 5.7 out of a maximum of 7, reflecting a moderate quality of life. The most affected areas were weakness and low energy, systemic symptom dysfunction, and psycho-emotional symptoms. Also, patients with compensated liver cirrhosis in advanced stages had lower scores for activities of daily living and energy level compared to those presenting with mild liver fibrosis (*p* < 0.05). This emphasizes that regardless of the stage of the disease, it can affect the patient’s quality of life, affecting their lifestyle, diet, and health [79]. Taru and co-workers implemented a Romanian-adapted appetite questionnaire called “CLDQ-RO” to assess appetite in a population of 231 Romanians, 35.5% of whom had compensated liver cirrhosis and 50.2% had decompensated liver cirrhosis. This questionnaire was also structured by domains such as the presence of abdominal symptoms, lethargy, systemic symptoms, emotional function, and concerns about the impact of the disease. It also used Likert scales to interpret patients’ responses (1 to 7), but this test implemented 29 items, whereas the present study implemented 36 items. Thus, following the answers given by the participants, there was a significant decrease in the score with the progression of the pathology, which indicates that the severity of the pathology leads to significant changes in the health and quality of life of the patient with liver cirrhosis. The most significant health change reported by the majority of participants was abdominal discomfort, specifically the presence of ascites, which was statistically significant (*p* = 0.004) [80]. Younossi et al. administered an appetite questionnaire (CLDQ) to several groups of patients with non-alcoholic steatohepatitis as the cause of their disease, including a subgroup of patients with compensated liver cirrhosis (about 23% of the group). The application of this instrument resulted in low quality of life scores, especially for those presenting with complex symptoms, but did not specify the exact scores for liver cirrhosis cases. The questionnaire assessed various symptoms: abdominal (pain, fullness, discomfort), physical (fatigue, physical activity), systemic (fever, muscle pain), psycho-emotional (anxiety, depression), and personal (concern about the general state of the disease, impact of the disease). Thus, the participants’ most commonly reported symptoms were chronic fatigue, abdominal pain and discomfort, pruritus, decreased appetite, insomnia, and lethargy (mainly caused by the presence of hepatic encephalopathy) [81].

The use of various types of questionnaires and clinical scores allows for a meaningful analysis of the complications associated with this disease. These scoring systems play a critical role in guiding clinical decision-making, especially in identifying the risk of complications. Integrating these established scores with emerging biomarkers may enhance their predictive accuracy and support the development of composite models. Such comprehensive risk stratification approaches are better suited to reflect the complexity of cirrhosis progression and may ultimately lead to improved patient outcomes [82].

## 5. Conclusions

The main aim of this study was to analyze the impact of nutrition and its role in patients with liver cirrhosis, thus demonstrating its importance in treatment plans. The results of the applied questionnaires, the “CNAQ” and “CLDQ-NASH”, revealed a low appetite and significantly impaired quality of life, demonstrated by scores below 28 points in most of the patients for appetite and below 4 for quality of life. The analysis of the 24 h recall revealed an unbalanced diet in most participants, with a predominant intake of pork and beef, omelets, bread, and traditional foods such as soups, and a low intake of white meat, fish, legumes, and dietary fiber. A small number of the participants complied with international dietary recommendations (those that were analyzed in this study were ESPEN, EALS, and AASLD). In particular, imbalances were reported in meal frequency, protein intake, evening snacks, and dietary diversity. The areas of impact that were specified by most of the patients were abdominal symptoms, psycho-emotional well-being, daily activities, and the impact of the disease on health.

The limitations of the study include the relatively low number of participants, limited diversity, a possible division of the group according to a “Child–Pugh” score, and variations in the etiologic cause of the disease. This study was observational and relied on patients’ self-reported responses, with the only nutritional guidance being the 24 h dietary booster, where portions were illustrated in pictures and tables. A limitation in this regard is the challenge of implementing a nutritional intervention over a long period of time, which would require repeated application of the boosters and questionnaires. The patients were willing to participate in the study, which confirms an interest in understanding the role of nutritional management in this pathology; therefore, it is recommended to develop nutritional protocols and include a dietician in the multidisciplinary treatment team for liver cirrhosis.

## Figures and Tables

**Table 1:** Age distribution of participants (%).

Age	No. Participants	Percent	Age	No. Participants	Percent
29	1	2%	59	4	8%
42	1	2%	60	6	12%
43	1	2%	61	3	6%
44	1	2%	62	1	2%
48	2	4%	63	3	6%
49	3	6%	64	1	2%
50	4	8%	65	2	4%
54	1	2%	68	1	2%
55	2	4%	69	2	4%
56	1	2%	70	7	14%
57	1	2%	71	2	4%

**Table 2:** Sample distribution by BMI (kg/m^2^) and weight.

No	BMI (kg/m^2^)	Body Mass Classification	No	BMI (kg/m^2^)	Body Mass Classification
1	33.46	O1	26	35.64	O2
2	25.63	overweight	27	32.44	O1
3	19.37	normal range	28	34.37	O2
4	25.2	overweight	29	30.37	O1
5	23.05	normal range	30	32.81	O1
6	25.9	overweight	31	28.71	overweight
7	29.93	overweight	32	30.45	O1
8	33.87	O1	33	29.90	overweight
9	32.59	O1	34	31.25	O1
10	28.18	overweight	35	26.94	overweight
11	26.02	overweight	36	34.58	O1
12	31.79	O1	37	27.65	overweight
13	31.4	O1	38	23.87	normal range
14	32.52	O1	39	30.50	O1
15	35.91	O2	40	31.48	O1
16	31.79	O1	41	26.41	overweight
17	29.94	overweight	42	31.48	O1
18	25	overweight	43	29.90	overweight
19	26.98	overweight	44	32.63	O1
20	27.68	overweight	45	29.11	overweight
21	30.86	O1	46	25.60	overweight
22	18.75	normal range	47	26.33	overweight
23	31.49	O1	48	27.33	overweight
24	32.92	O1	49	28.42	overweight
25	27.34	overweight	50	31.43	O1

O1—obesity type 1; O2—obesity type 2.

## Data Availability

The original contributions presented in this study are included in the article. Further inquiries can be directed to the corresponding author.

## Abbreviations

The following abbreviations are used in this manuscript:

AASLD	American Association for the Study of Liver Diseases
ALT	Alanine Aminotransferase
AST	Aspartate Aminotransferase
CLDQ-NASH	The Chronic Liver Disease Questionnaire for Nonalcoholic Steatohepatitis
CNAQ	Council of Nutrition Appetite Questionnaire
CNS	Central nervous system
EALS	European Association for the Study of the Liver Guidelines
ESPEN	European Society for Clinical Nutrition and Metabolism
GBD	Global Burden of Diseases, Injuries, and Risk Factors Study
GPP	Good practice point
MELD	The Model for End-Stage Liver Disease
NAFLD	Non-alcoholic fatty liver disease

## References

[1] Pimpin L., Cortez-Pinto H., Negro F., Corbould E., Lazarus J.V., Webber L., Sheron N., The Members of the EASL HEPAHEALTH Steering Committee (2018). Burden of liver disease in Europe: Epidemiology and analysis of risk factors to identify prevention policies. J. Hepatol..

[2] Tapper E.B., Parikh N.D. (2018). Mortality due to cirrhosis and liver cancer in the United States, 1999–2016: Observational study. BMJ.

[3] Safiri S., Sepanlou S.G., Ikuta K.S., Bisignano C., Salimzadeh H., Delavari A., Ansari R., Roshandel G., Merat S., Fitzmaurice C. (2019). The global, regional, and national burden of colorectal cancer and its attributable risk factors in 195 countries and territories, 1990–2017: A systematic analysis for the Global Burden of Disease Study 2017. Lancet Gastroenterol. Hepatol..

[4] Cirrhosis: Diagnosis and Management—PubMed. https://pubmed.ncbi.nlm.nih.gov/31845776/.

[5] Tapper E.B., Parikh N.D. (2023). Diagnosis and Management of Cirrhosis and Its Complications: A Review. JAMA.

[6] Muir A.J. (2015). Understanding the Complexities of Cirrhosis. Clin. Ther..

[7] Merli M., Berzigotti A., Zelber-Sagi S., Dasarathy S., Montagnese S., Genton L., Plauth M., Parés A. (2019). EASL Clinical Practice Guidelines on nutrition in chronic liver disease. J. Hepatol..

[8] Santangeli E., Abbati C., Chen R., Di Carlo A., Leoni S., Piscaglia F., Ferri S. (2024). Pathophysiological-Based Nutritional Interventions in Cirrhotic Patients with Sarcopenic Obesity: A State-of-the-Art Narrative Review. Nutrients.

[9] Traub J., Reiss L., Aliwa B., Stadlbauer V. (2021). Malnutrition in patients with liver cirrhosis. Nutrients.

[10] Naqvi I.H., Mahmood K., Salekeen S., Akhter S.T. (2013). Determining the frequency and severity of malnutrition and correlating it with the severity of liver cirrhosis. Turk. J. Gastroenterol..

[11] Nutritional Status and Its Impact on Clinical Outcomes for Patients Admitted to Hospital with Cirrhosis—PubMed. https://pubmed.ncbi.nlm.nih.gov/27266216/.

[12] Maharshi S., Sharma B.C., Srivastava S. (2015). Malnutrition in cirrhosis increases morbidity and mortality. J. Gastroenterol. Hepatol..

[13] Ribeiro H.S., Maurício S.F., da Silva T.A., de Vasconcelos Generoso S., Lima A.S., Correia M.I.T.D. (2018). Combined nutritional assessment methods to predict clinical outcomes in patients on the waiting list for liver transplantation. Nutrition.

[14] Ferreira L.G., Martins A.I.F., Cunha C.E., Anastácio L.R., Lima A.S., Correia M.I.T.D. (2013). Negative energy balance secondary to inadequate dietary intake of patients on the waiting list for liver transplantation. Nutrition.

[15] Izbéki F., Kiss I., Wittmann T., Várkonyi T.T., Légrády P., Lonovics J. (2002). Impaired accommodation of proximal stomach in patients with alcoholic liver cirrhosis. Scand. J. Gastroenterol..

[16] Aprile L.R.O., Meneghelli U.G., Martinelli A.L.C., Monteiro C.R. (2002). Gastric motility in patients with presinusoidal portal hypertension. Am. J. Gastroenterol..

[17] Aqel B.A., Scolapio J.S., Dickson R.C., Burton D.D., Bouras E.P. (2005). Contribution of ascites to impaired gastric function and nutritional intake in patients with cirrhosis and ascites. Clin. Gastroenterol. Hepatol..

[18] Grüngreiff K., Reinhold D., Wedemeyer H. (2016). The role of zinc in liver cirrhosis. Ann. Hepatol..

[19] Lanspa S.J., Chan A.T.H., Bell J.S., Go V.L.W., Dickson E.R., Dimagno E.P. (1985). Pathogenesis of steatorrhea in primary biliary cirrhosis. Hepatology.

[20] Hofmann A.F. (1999). The continuing importance of bile acids in liver and intestinal disease. Arch. Intern. Med..

[21] Glucose Intolerance Insulin Resistance in Patients with Liver Disease. II. A Study of Etiologic Factors and Evaluation of Insulin Actions—PubMed. https://pubmed.ncbi.nlm.nih.gov/5506964/.

[22] Müller M.J., Böttcher J., Selberg O., Weselmann S., Böker K.H., Schwarze M., Mühlen A.v.Z., Manns M.P. (1999). Hypermetabolism in clinically stable patients with liver cirrhosis. Am. J. Clin. Nutr..

[23] Nakaya Y., Harada N., Kakui S., Okada K., Takahashi A., Inoi J., Ito S. (2002). Severe catabolic state after prolonged fasting in cirrhotic patients: Effect of oral branched-chain amino-acid-enriched nutrient mixture. J. Gastroenterol..

[24] E Owen O., A Reichle F., Mozzoli M.A., Kreulen T., Patel M.S., Elfenbein I.B., Golsorkhi M., Chang K.H., Rao N.S., Sue H.S. (1981). Hepatic, gut, and renal substrate flux rates in patients with hepatic cirrhosis. J. Clin. Investig..

[25] Petersen K.F., Krssak M., Navarro V., Chandramouli V., Hundal R., Schumann W.C., Landau B.R., Shulman G.I. (1999). Contributions of net hepatic glycogenolysis and gluconeogenesis to glucose production in cirrhosis. Am. J. Physiol. Metab..

[26] Yeung R.T.T., Wang C.C.L. (1974). A study of carbohydrate metabolism in postnecrotic cirrhosis of liver. Gut.

[27] Marchesini G., Bianchi G., Lucidi P., Villanova N., Zoli M., De Feo P. (2004). Plasma Ghrelin Concentrations, Food Intake, and Anorexia in Liver Failure. J. Clin. Endocrinol. Metab..

[28] Iwasa M., Iwata K., Hara N., Hattori A., Ishidome M., Sekoguchi-Fujikawa N., Mifuji-Moroka R., Sugimoto R., Fujita N., Kobayashi Y. (2013). Nutrition therapy using a multidisciplinary team improves survival rates in patients with liver cirrhosis. Nutrition.

[29] Reuter B., Shaw J., Hanson J., Tate V., Acharya C., Bajaj J.S. (2019). Nutritional Assessment in Inpatients with Cirrhosis Can Be Improved After Training and Is Associated with Lower Readmissions. Liver Transplant..

[30] Sam J., Nguyen G.C. (2009). Protein-calorie malnutrition as a prognostic indicator of mortality among patients hospitalized with cirrhosis and portal hypertension. Liver Int..

[31] Nielsen K., Kondrup J., Martinsen L., Stilling B., Wikman B. (1993). Nutritional assessment and adequacy of dietary intake in hospitalized patients with alcoholic liver cirrhosis. Br. J. Nutr..

[32] Ney M., Abraldes J.G., Ma M., Belland D., Harvey A., Robbins S., Heyer V.D., Tandon P. (2015). Insufficient Protein Intake Is Associated with Increased Mortality in 630 Patients with Cirrhosis Awaiting Liver Transplantation. Nutr. Clin. Pract..

[33] Plank L.D., Gane E.J., Peng S., Muthu C., Mathur S., Gillanders L., McIlroy K., Donaghy A.J., McCall J.L. (2008). Nocturnal nutritional supplementation improves total body protein status of patients with liver cirrhosis: A randomized 12-month trial. Hepatology.

[34] Swart G.R., Zillikens M.C., Van Vuure J.K., Van den Berg J.W.O. (1989). Effect of a late evening meal on nitrogen balance in patients with cirrhosis of the liver. Br. Med. J..

[35] Tsien C.D., Mccullough A.J., Dasarathy S. (2012). Late evening snack: Exploiting a period of anabolic opportunity in cirrhosis. J. Gastroenterol. Hepatol..

[36] Guo Y.J., Tian Z.B., Jiang N., Ding X.L., Mao T., Jing X. (2018). Effects of late evening snack on cirrhotic patients: A systematic review and meta-analysis. Gastroenterol. Res. Pract..

[37] Food Groups Intake of Cirrhotic Patients, Comparison with the Nutritional Status and Disease Stage—PubMed. https://pubmed.ncbi.nlm.nih.gov/31528306/.

[38] Haberl J., Zollner G., Fickert P., Stadlbauer V. (2018). To salt or not to salt?—That is the question in cirrhosis. Liver Int..

[39] Gu X.B., Yang X.J., Zhu H.Y., Xu B.Y. (2012). Effect of a diet with unrestricted sodium on ascites in patients with hepatic cirrhosis. Gut Liver.

[40] Putadechakum S., Klangjareonchai T., Soponsaritsuk A., Roongpisuthipong C. (2012). Nutritional status assessment in cirrhotic patients after protein supplementation. ISRN Gastroenterol..

[41] Cunha L., Nono M.H., Guibert A.L., Nidegger D., Beau P., Beauchant M. (2004). Effects of prolonged oral nutritional support in malnourished cirrhotic patients: Results of a pilot study. Gastroenterol. Clin. Biol..

[42] Ney M., Vandermeer B., Van Zanten S.J.V., Ma M.M., Gramlich L., Tandon P. (2013). Meta-Analysis: Oral or enteral nutritional supplementation in cirrhosis. Aliment. Pharmacol. Ther..

[43] Maharshi S., Sharma B.C., Sachdeva S., Srivastava S., Sharma P. (2016). Efficacy of Nutritional Therapy for Patients with Cirrhosis and Minimal Hepatic Encephalopathy in a Randomized Trial. Clin. Gastroenterol. Hepatol..

[44] Norman K., Kirchner H., Freudenreich M., Ockenga J., Lochs H., Pirlich M. (2008). Three month intervention with protein and energy rich supplements improve muscle function and quality of life in malnourished patients with non-neoplastic gastrointestinal disease-A randomized controlled trial. Clin. Nutr..

[45] Bering T., Diniz K.G., Coelho M.P.P., Vieira D.A., Soares M.M.S., Kakehasi A.M., Correia M.I.T., Teixeira R., Queiroz D.M., Rocha G.A. (2018). Association between pre-sarcopenia, sarcopenia, and bone mineral density in patients with chronic hepatitis C. J. Cachexia Sarcopenia Muscle.

[46] Carey E.J., Lai J.C., Wang C.W., Dasarathy S., Lobach I., Montano-Loza A.J., Dunn M.A. (2017). The Fitness, Life Enhancement, and Exercise in Liver Transplantation Consortium. A multicenter study to define sarcopenia in patients with end-stage liver disease. Liver Transplant..

[47] Chang K.V., De Chen J., Wu W.T., Huang K.C., Lin H.Y., Han D.S. (2019). Is sarcopenia associated with hepatic encephalopathy in liver cirrhosis? A systematic review and meta-analysis. J. Formos. Med. Assoc..

[48] Chinnaratha M.A., Chaudhary S., Doogue M., Mccormick R.J., Woodman R.J., Wigg A.J. (2015). Prevalence of hepatic osteodystrophy and vitamin D deficiency in cirrhosis. Intern. Med. J..

[49] Hanai T., Shiraki M., Nishimura K., Ohnishi S., Imai K., Suetsugu A., Takai K., Shimizu M., Moriwaki H. (2015). Sarcopenia impairs prognosis of patients with liver cirrhosis. Nutrition.

[50] Bunchorntavakul C., Reddy K.R. (2019). Review article: Malnutrition/sarcopenia and frailty in patients with cirrhosis. Aliment. Pharmacol. Ther..

[51] Kato M., Miwa Y., Tajika M., Hiraoka T., Muto Y., Moriwaki H. (1998). Preferential Use of Branched-Chain Amino Acids as an Energy Substrate in Patients with Liver Cirrhosis. Intern. Med..

[52] Abuelazm M., Fares A., Elhady M.M., Amin A.M., Khan U., Gowaily I., Jaber F. (2025). Branched-Chain Amino Acid Supplements for Sarcopenia in Liver Cirrhosis: A Systematic Review and Meta-analysis. J. Clin. Exp. Hepatol..

[53] Yamauchi M., Takeda K., Sakamoto K., Ohata M., Toda G. (2001). Effect of oral branched chain amino acid supplementation in the late evening on the nutritional state of patients with liver cirrhosis. Hepatol. Res..

[54] Nakaya Y., Okita K., Suzuki K., Moriwaki H., Kato A., Miwa Y., Shiraishi K., Okuda H., Onji M., Kanazawa H. (2007). BCAA-enriched snack improves nutritional state of cirrhosis. Nutrition.

[55] Muto Y., Sato S., Watanabe A., Moriwaki H., Suzuki K., Kato A., Kato M., Nakamura T., Higuchi K., Nishiguchi S. (2006). Overweight and obesity increase the risk for liver cancer in patients with liver cirrhosis and long-term oral supplementation with branched-chain amino acid granules inhibits liver carcinogenesis in heavier patients with liver cirrhosis. Hepatol. Res..

[56] Improvement of Hepatic Encephalopathy Using a Modified High-Calorie High-Protein Diet—PubMed. https://pubmed.ncbi.nlm.nih.gov/16200232/.

[57] Amodio P., Bemeur C., Butterworth R., Cordoba J., Kato A., Montagnese S., Uribe M., Vilstrup H., Morgan M.Y. (2013). The nutritional management of hepatic encephalopathy in patients with cirrhosis: International society for hepatic encephalopathy and nitrogen metabolism consensus. Hepatology.

[58] Merli M., Iebba V., Giusto M. (2015). What is new about diet in hepatic encephalopathy. Metab. Brain Dis..

[59] Plauth M., Cabré E., Riggio O., Assis-Camilo M., Pirlich M., Kondrup J., Ferenci P., Holm E., Dahl S.V., Müller M. (2006). ESPEN Guidelines on Enteral Nutrition: Liver disease. Clin. Nutr..

[60] Kearns P.J., Young H., Garcia G., Blaschke T., O’HAnlon G., Rinki M., Sucher K., Gregory P. (1992). Accelerated improvement of alcoholic liver disease with enteral nutrition. Gastroenterology.

[61] Baltz J.G., Argo C.K., Al-Osaimi A.M.S., Northup P.G. (2010). Mortality after percutaneous endoscopic gastrostomy in patients with cirrhosis: A case series. Gastrointest. Endosc..

[62] Cabré E., Rodríguez-Iglesias P., Caballería J., Quer J.C., Sánchez-Lombraña J.L., Parés A., Papo M., Planas R., Gassull M.A. (2000). Short- and long-term outcome of severe alcohol-induced hepatitis treated with steroids or enteral nutrition: A multicenter randomized trial. Hepatology.

[63] Wilson M.M., Thomas D.R., Rubenstein L.Z., Chibnall J.T., Anderson S., Baxi A., Diebold M.R., Morley J.E. (2005). Appetite assessment: Simple appetite questionnaire predicts weight loss in community-dwelling adults and nursing home residents. Am. J. Clin. Nutr..

[64] Graur M. (2006). Ghid de Alimentație Sănătoasă.

[65] Younossi Z.M., Stepanova M., Younossi I., Racila A. (2019). Validation of Chronic Liver Disease Questionnair for Nonalcoholic Steatohepatitis in Patients with Biopsy-Proven Nonalcoholic Steatohepatitis. Clin. Gastroenterol. Hepatol..

[66] Vasilachi G., Vasilachi A. (2016). Alimentația Omului Sănătos și a Omului Bolnav (Recomandări Pentru Cele Mai Diverse Cazuri de Boală).

[67] https://www.nutritionvalue.org/.

[68] Carbs C.C., Carb C. (1882). Calorie Counter.

[69] Wang T., Shen J. (2018). Usefulness of simplified nutritional appetite questionnaire (SNAQ) in appetite assesment in elder patients with liver cirrhosis. J. Nutr. Health Aging.

[70] Rudzińska A., Czesak J., Wieczorek-Stawińska W., Gąsowski J., Piotrowicz K. (2024). Taste assessment as a part of geriatric nutritional care: Potential implications for clinical practice. Clin. Nutr. Open Sci..

[71] Towey J., Ngonadi C., Greig C., Armstrong M. (2024). O4 Impact of hepatic encephalopathy in advanced chronic liver disease on appetite and nutritional intake: An exploratory prospective observational UK study. Gut.

[72] Georgiou A., Yannakoulia M., Papatheodoridis G.V., Deutsch M., Alexopoulou A., Vlachogiannakos J., Ioannidou P., Papageorgiou M.V., Voulgaris T., Papadopoulos N. (2021). Assessment of dietary habits and the adequacy of dietary intake of patients with cirrhosis-the KIRRHOS study. Clin. Nutr..

[73] Sharma P., Gupta C., Kumar A., Arora A., Anikhindi S.A., Singla V., Bansal N., Jasrotia S. (2021). Nutritional assessment and factors affecting dietary intake in patients with cirrhosis: A single-center observational study. Nutrition.

[74] Pham D.M., Nguyen T.D. (2023). Nutritional Status, Dietary and Nutrition Impact Symptoms of Cirrhotic Patients. Tạp Chí Dinh Dưỡng và Thực Phẩm.

[75] Ferreira S.C., Cardoso Ade S.R., Machado Ade A.S., Anastácio L.R. (2024). Effect of a 12-week nutritional intervention in the food intake of patients on the waiting list for liver transplantation: A secondary analysis of a randomized controlled trial. Clin. Nutr..

[76] Trang T.N., Bùi A., Nguyen L., Chinh T., Thi P., Oanh V., Shigure Y. (2021). Nutritional Status and Nutritional Practice of Cirrhotic Patients at Hanoi Medical University Hospital, 2020. Asian J. Diet..

[77] Mayank Jain J.K., Vargese J., Srinivasan V., Harika K., Michael T., Venkataraman J. (2018). Health-related quality of life in liver cirrhosis patients using SF-36 and CLDQ questionnaires. Clin. Exp. Hepatol..

[78] Alavinejad P., Hajiani E., Danyaee B., Morvaridi M. (2019). The effect of nutritional education and continuous monitoring on clinical symptoms, knowledge, and quality of life in patients with cirrhosis. Gastroenterol. Hepatol. Bed Bench.

[79] Yamamura S., Nakano D., Hashida R., Tsutsumi T., Kawaguchi T., Okada M., Isoda H., Takahashi H., Matsuse H., Eguchi Y. (2021). Patient-reported outcomes in patients with non-alcoholic fatty liver disease: A narrative review of Chronic Liver Disease Questionnaire-non-alcoholic fatty liver disease/non-alcoholic steatohepatitis. J. Gastroenterol. Hepatol..

[80] Taru V., Indre M.G., Ignat M.D., Forgione A., Racz T., Olar B.A., Farcau O., Chereches R., Stefanescu H., Procopet B. (2021). Validation and performance of chronic liver disease questionnaire (Cldq-ro) in the romanian population. J. Gastrointest. Liver Dis..

[81] Younossi Z.M., Stepanova M., Younossi I., Racila A. (2025). Validation of the Chronic Liver Disease Questionnaire for MASH (CLDQ-MASH). JHEP Rep..

[82] Scarlata G.G.M., Ismaiel A., Gambardella M.L., Leucuta D.C., Luzza F., Dumitrascu D.L., Abenavoli L. (2024). Use of Non-Invasive Biomarkers and Clinical Scores to Predict the Complications of Liver Cirrhosis: A Bicentric Experience. Medicina.

